# Horizontal transmission of symbiotic bacteria and host selective sweep in the giant clam *Tridacna crocea*

**DOI:** 10.1093/ismeco/ycaf037

**Published:** 2025-03-02

**Authors:** Cong Liu, Jian Zhang, Qiqi Li, Yuehuan Zhang, Si Zhang, Ziniu Yu, Jun Li, Jie Li

**Affiliations:** CAS Key Laboratory of Tropical Marine Bio-Resources and Ecology, South China Sea Institute of Oceanology, Chinese Academy of Sciences, Guangzhou 510301, China; University of Chinese Academy of Sciences, Beijing 100049, China; CAS Key Laboratory of Tropical Marine Bio-Resources and Ecology, South China Sea Institute of Oceanology, Chinese Academy of Sciences, Guangzhou 510301, China; CAS Key Laboratory of Tropical Marine Bio-Resources and Ecology, South China Sea Institute of Oceanology, Chinese Academy of Sciences, Guangzhou 510301, China; CAS Key Laboratory of Tropical Marine Bio-Resources and Ecology, South China Sea Institute of Oceanology, Chinese Academy of Sciences, Guangzhou 510301, China; CAS Key Laboratory of Tropical Marine Bio-Resources and Ecology, South China Sea Institute of Oceanology, Chinese Academy of Sciences, Guangzhou 510301, China; Sanya National Marine Ecosystem Research Station, Chinese Academy of Sciences, Sanya 572000, China; CAS Key Laboratory of Tropical Marine Bio-Resources and Ecology, South China Sea Institute of Oceanology, Chinese Academy of Sciences, Guangzhou 510301, China; CAS Key Laboratory of Tropical Marine Bio-Resources and Ecology, South China Sea Institute of Oceanology, Chinese Academy of Sciences, Guangzhou 510301, China; Sanya National Marine Ecosystem Research Station, Chinese Academy of Sciences, Sanya 572000, China; CAS Key Laboratory of Tropical Marine Bio-Resources and Ecology, South China Sea Institute of Oceanology, Chinese Academy of Sciences, Guangzhou 510301, China; Sanya National Marine Ecosystem Research Station, Chinese Academy of Sciences, Sanya 572000, China

**Keywords:** bacterial communities, *Tridacna crocea*, horizontal transmission, early life stages

## Abstract

Giant clams, with their significant ecological importance, depend on associated bacteria for their health and development, yet the transmission modes and succession of community dynamics of these bacteria remain poorly understood. This study employed 16S rRNA gene sequencing and microscopy to investigate the transmission and community dynamics of symbiotic bacteria in the giant clam *Tridacna crocea* during early developmental stages (fertilized eggs, blastocyst, D-larvae, and pediveliger larvae). Fluorescence in situ hybridization and transmission electron microscopy did not detect internal symbiotic bacteria in fertilized eggs and adult gonad gametes, but scanning electron microscopy revealed microbial structures on egg surface microvilli, suggesting their role as microbial carriers. 16S rRNA sequencing confirmed microbial presence in fertilized eggs, indicating bacterial acquisition via external vertical transmission (adherence to microvilli) or horizontal transmission. Given the lack of internalized bacteria in reproductive organs, we prefer to classify the symbiotic bacteria acquisition as horizontal transmission. Microbial community analysis showed that *T. crocea* acquired a significant portion of its microbiome from seawater throughout its development. Before reaching the pediveliger stage, the bacterial community composition closely resembled that of the surrounding seawater, primarily featuring the family *Rhodobacteraceae*. As *T. crocea* matured, the host’s selective pressure increased (e.g. deterministic assembly), which simplified the microbial community and reduced diversity. During the pediveliger stage, the genus *Endozoicomonas* became dominant, forming a large proportion of the bacterial community within the gonads. This highlights the ecological significance of host–microbe interactions in maintaining biodiversity and driving ecosystem stability through dynamic community assembly processes.

## Introduction

It is widely known that marine bivalves have symbiotic interactions with microorganisms, but the process and mechanism by which hosts establish a diverse and functional microbial composition has long confused researchers. Giant clams are essential to coral reef ecosystems due to their significant economic and ecological roles [1]. It has been reported that adult giant clams harbor diverse and functionally rich microbial communities [2, 3], with these symbiotic microbes potentially closely linked to the host’s physiological functions, growth, and susceptibility to diseases or mortality [4–8]. As broadcast spawners, these clams release their gametes into the water column [9–11]. The early life stages of giant clams encompass fertilized egg, blastocyst, gastrulation, trochophore, D-larvae, pediveliger larvae, and ultimately spat [10, 11]. During this developmental process, the mechanisms by which giant clams assemble such complex and stable symbiotic bacterial communities remain unknown.

The initial colonization by microbes plays a pivotal role in shaping the developmental dynamics of the entire microbial community, which is critical for preserving the health of symbiotic systems [12, 13]. Hosts typically employ various transmission modes for their symbionts, including strict vertical transmission, horizontal transmission from the environment, and mixed modes [14]. In the case of strict vertical transmission, symbionts are passed from the symbiont housing organ to the female gonads before reproduction, ensuring symbiotic descendants. On the other hand, horizontal transmission occurs when host reproduction yields aposymbiotic descendants, which acquire symbiotic bacteria anew with each host generation. Mixed modes, which incorporate elements of both vertical and horizontal transmission, are also observed [14]. Symbionts transmitted vertically reside within the host throughout their life cycle and do not compete with environmental bacteria, potentially experiencing population bottlenecks, leading to genome reduction and functional loss [14–16]. In contrast, horizontal transmission promotes genetic exchange with the environmental microbiota, enhancing the host’s ability to adapt to environmental changes [17]. Hence, microbiota acquired through vertical transmission are typically more stable, while those obtained via horizontal transmission are more reliant on the environmental microbiota [17]. Different hosts may adopt distinct microbial symbiont transmission strategies. To rigorously assess the mode of transmission of symbiotic bacteria in hosts, methods such as transmission electron microscopy (TEM) and fluorescence in situ hybridization (FISH) are employed. These techniques enable detailed visualization of bacterial localization and provide insights into whether bacteria are vertically or horizontally transmitted, helping to resolve the complex dynamics of microbial community assembly in symbiotic hosts [18–20].

Apart from the initial colonizinge microbiota, symbiotic microbial communities within hosts may undergo stage-specific changes during different developmental stages [21, 22]. Marine animals, such as bivalves, may alter their microbial community composition through recruitment and enrichment, acquiring microbes from the surrounding environment to support host health and development [23–25]. However, the assembly of microbial communities is influenced by both host-specific factors and the surrounding environment. To dissect this intricate issue, the use of null models can help simplify this complex problem, which can quantify the underlying mechanisms of microbial community assembly. Stochastic and deterministic processes represent two ecological processes that shape community assembly [26]. Deterministic processes shape species presence, absence, and relative abundances through abiotic and biotic environmental filtering, while stochastic processes involve random changes in species abundances driven by factors such as ecological drift and probabilistic dispersal [27]. Previous studies have applied the null model to investigate the internal driving forces of deterministic processes in stage-specific community types during the early development of Kumamoto oyster *Crassostrea sikamea* [21, 22]. However, the intricate dynamics and assembly processes of bacteria during the early life stages of giant clams remain poorly understood.

Here, we focus on *Tridacna crocea*, commonly known as the boring giant clam, which plays a key ecological role within coral reef ecosystems. Among the giant clam species, *T. crocea* is notable for its small size, rapid sexual maturity, and broad geographical distribution across the Indo-Malay Archipelago [28]. These traits make *T. crocea* an ideal model for studying microbial transmission and community dynamics during host development. In this study, we utilized an array of methodologies, including FISH, TEM, hematoxylin and eosin (HE) staining, and scanning electron microscopy (SEM), to analyze and visualize the transmission modes of microbial communities within *T. crocea.* Additionally, using 16S rRNA gene sequencing, we analyzed the succession, source, and assembly mechanisms of bacterial community dynamics at fine-scale developmental stages (adult gonad, fertilized eggs, blastocyst, D-larvae, and pediveliger larvae). Bacterial assemblages associated with *T. crocea*’s early life stages were compared to those found in adult gonads and seawater. This comprehensive study will lay a theoretical foundation for a deeper understanding of the interaction between giant clams and microorganisms, as well as the mechanisms involved in their symbiosis.

## Materials and methods

### Giant clam spawning, rearing, and sample collection

Giant clam *T. crocea* was temporarily bred at the tropical marine biological research station in Sanya, Hainan Province, employing microfluidic water for gonadotrophic maturation from 1 March 2022, until spawning on 20 April 2022. Three randomly selected *T. crocea* individuals were induced to spawn by the injection of 1 mL of a serotonin solution (1 mg/mL, Sigma H9523-25MG) into their adductor muscle [29, 30]. Following the injection, each *T. crocea* was transferred to a plastic bucket containing 30 L of fresh 10 μm filtered seawater (FSW). After 3–6 min, the *T. crocea* initiated releasing sperm, typically lasting for approximately 10 min. Subsequently, they were transferred to a plastic bucket filled with 50 L of FSW, and the release of eggs commenced after 10–12 min, lasting from 30 min to 1 h. Fertilization from different individuals was carried out by combining the collected eggs and viable sperm at a ratio of 50:1–100:1. Following successful fertilization, the incubation water was gently agitated, and the fertilized eggs (0 h) were transferred into the incubation tank. The detailed breeding procedures can be found in the Supplementary methods.

**Figure 1:**
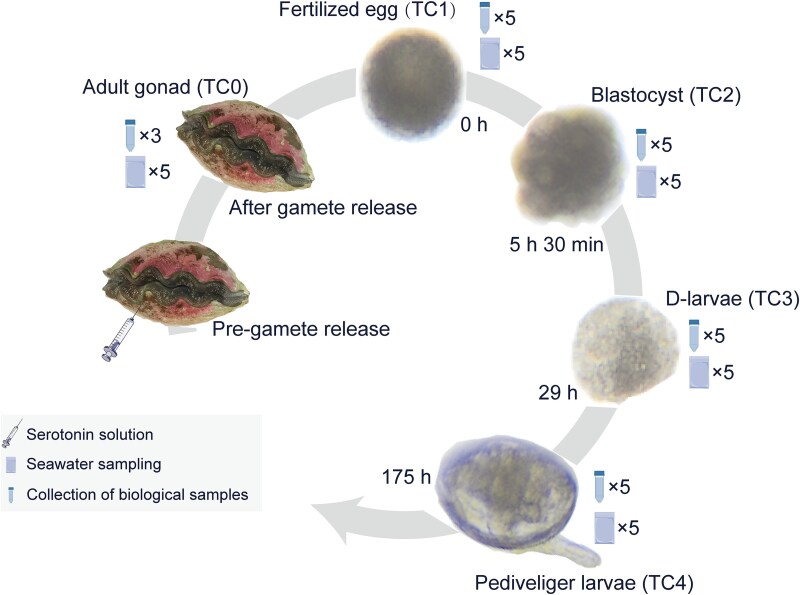
Illustration of the experimental design for spawning, sampling, and rearing of *Tridacna crocea*.

The adult gonads (TC0) were collected from each adult *T. crocea* after spawning. Early developmental stages, including fertilized eggs (TC1), blastocyst (TC2), D-larvae (TC3, before feeding algae), and pediveliger larvae (TC4), were sampled using a 300-mesh screen for five replicates. All samples were rinsed with autoclaved 0.22 μm-filtered FSW to remove loosely attached microbes before DNA extraction or fixation for microscopy. Samples (~30 mg) intended for DNA extraction were stored in 99% ethanol at −80 °C. Samples for FISH microscopy were fixed in 4% paraformaldehyde (PFA) and left at 4 °C for ~10 h, and then washed in phosphate buffered saline (PBS) and stored at −20 °C in 70% ethanol–PBS solution. Additionally, the seawater (1 L, five replicates per sample) was collected by sterile sampling bag for microbial community analysis from each tank at every sampling time point (Fig. 1). The seawater was filtered through 0.22 μm polyvinylidene fluoride (PVDF) membranes (GVWP04700, Durapore, Merck Millipore, Ireland) using a peristaltic pump, and the filters were stored at −80 °C. For TEM analysis, samples were fixed in 2.5% glutaraldehyde (EM Grade) and stored at 4 °C until further processing. Sample preparation for SEM was conducted following the same method as that for TEM. For microscopic experiments, at least 50 individuals are required for each stage of the sample.

### Histology and fluorescence in situ hybridization microscopy on slides

Histological processing was performed after modifying the method described in the reference [31]. Briefly, following fixation, D-larvae and pediveliger larvae were decalcified in 0.5 M EDTA at 4 °C, with the solution being changed every 24 h until complete decalcification was achieved, typically within two days for pediveliger larvae and one day for D-larvae. Following decalcification, these larvae, along with fertilized eggs and blastocyst were embedded in 2% agar. Then, together with the fixed gonad samples, they were rinsed, dehydrated, and wax-leached using a dehydrator (DIAPATH, Donatello). They were then embedded in paraffin wax using an embedding machine (Wuhan Junjie Electronics Co., Ltd, JB-P5), and sectioned into slices of 4 μm thickness. Sections were dewaxed at 60 °C before histological processing. Subsequently, tissue sections were stained with a HE staining kit obtained from Boster Biological Technology, Ltd (Wuhan, China), and images were captured using a microscope (Leica DM 2500).

For FISH [31, 32], paraffin-embedded tissue sections (4 μm) were prepared for hybridization using Cy3-labeled EUB338 probes. The labeled NONEUB338 probe was served as a negative control. The detailed FISH experimental protocol is provided in the Supplementary methods.

### Transmission electron microscopy and scanning electron microscope

For TEM analysis, after fixation, D-larvae and pediveliger larvae were decalcified in 0.5 M EDTA at 4 °C, with the EDTA solution being refreshed every 24 h until decalcification was fully achieved, typically requiring two days for pediveliger larvae and one day for D-larvae. Subsequently, all tissue samples, encompassing gonads, fertilized eggs, blastocyst, D-larvae, and pediveliger larvae, were washed using 0.1 M phosphate buffer (PB, pH 7.4), postfixed with 1% OsO_4_ (Ted Pella Inc, 18 456), dehydrated, and embedded in EMBed 812 (SPI, 90529-77-4). After polymerization, the ultrathin sections (60–80 nm) were stained with 2% uranium acetate and 2.6% lead citrate, and images were acquired on a TEM (HITACHI HT7800).

For SEM analysis, after fixation, the fertilized eggs were washed with 0.1 M PB (pH 7.4), postfixed with 1% OsO_4_ (Ted Pella Inc., 18 456), and dehydrated. After critical point drying (Quorum K850), gold coating, and specimen mounting, the samples were examined using a SEM (HITACHI SU8100).

### DNA extraction, amplicon sequencing, and data processing

*Tridacna crocea* samples were washed with autoclave 1 × PBS buffer before DNA extraction. The samples from the early developmental stages were rapidly frozen in liquid nitrogen and subsequently homogenized using a sterile plastic pestle (JET BIOFIL, Guangzhou, China) in 1.5 mL tubes [33]. The gonads of *T. crocea* were homogenized by using the tissue grinder (Ti-PrP-02, Shanghai Jingxin Industrial Development Co., Ltd, China) under liquid nitrogen (cryomilling). Seawater samples’ filters were cut into pieces using sterilized surgical blades for DNA extraction. DNA extraction from homogenized tissues and seawater sample membranes was conducted using the DNeasy PowerSoil Pro Kits (Cat: 47016, QIAGEN) following the manufacturer’s instructions. DNA concentrations were measured by using a NanoDrop 2000 UV–vis spectrophotometer (Thermo Scientific, Wilmington, USA). The amplification of the 16S rRNA gene was conducted using the primers 27F [5′-AGRGTTYGATYMTGGCTCAG-3′] and 1492R [5′-RGYTACCTTGTTACGACTT-3′] [34]. Polymerase chain reaction assays were performed in triplicate for each sample, and amplicons were sequenced using the PacBio Sequel II System. Processed high-quality sequences were further processed using the DADA2 plugin [35] within QIIME2 [36] to generate amplicon sequence variants (ASVs). Detailed descriptions of the library preparation, sequencing, and bioinformatics pipeline are provided in the Supplementary methods.

### Bacterial community analysis

The Shannon index for alpha diversity was calculated from rarefied ASVs [37] using the vegan R package [38]. Statistical analyses (*t*-test) were performed using the “rstatix” and “ggpubr” packages in R [39], with * indicating *P* < .05 and ** indicating *P* < .01. For assessing beta diversity, principal coordinate analysis (PCoA) was performed using the Bray–Curtis dissimilarity metric using the vegan R package [40], and the result was statistically confirmed by Adonis. LEfSe software was used to perform linear discriminant analysis (LDA) effect size (LEfSe) analysis to identify potential microbial biomarkers through differential abundance analysis, with a LDA score threshold of >3.5. Statistical analyses and visualizations were performed using the ImageGP web service [41]. Source tracking analysis was performed using SourceTracker [42], where each sampling stage was identified as a sink, with fertilized eggs as the starting point, and seawater samples from the current time point and hosts from the previous period were treated as potential sources. Phylogenetic-bin-based null model analysis (iCAMP) was used to quantify the microbial community assembly process [43] using the R package iCAMP (1.5.12). Based on iCAMP, the quantitative results on community assembly processes containing heterogeneous selection (HeS), homogeneous selection (HoS), dispersal limitation (DL), homogenizing dispersal (HD), and undominated were obtained from the statistical perspective. The construction and visualization of the co-occurrence network were performed using the “ggClusterNet” (0.1.0) [44] package in R and Gephi (0.10.1) [45]. The top150 ASVs in terms of relative abundance in each group were selected for network construction, with low-abundance taxa filtered out to reduce noise, retaining only edges with a correlation coefficient *r* > 0.8 and *P* < .05. Keystone ASVs in the network were identified based on their within-module connectivity (*Zi*) and among-module connectivity (*Pi*). Nodes with *Zi* ≥ 2.5 or *Pi* ≥ 0.62 were classified as keystone ASVs. Using the “adonis” package in R, the permutational multivariate analysis of variance (PERMANOVA) was used to evaluate the effects of habitat (host, water), time, and their interactions on the variations in the bacterial community [46].

### Phylogenetic analysis of amplicon sequence variants affiliated with the genus *Endozoicomonas*

*Endozoicomonas* is a well-established and ecologically significant genus of bacteria commonly found in marine invertebrates [47]. Phylogenetic analysis of ASVs associated with the family *Endozoicomonadaceae* was conducted in this study. A total of 258 ASVs were identified as belonging to the family *Endozoicomonadaceae*. Notably, all members of this family were classified under the genus *Endozoicomonas*. Multiple sequence alignments were performed for the ASVs using CLUSTALW. Subsequently, phylogenetic trees were constructed using the neighbor-joining (NJ) method implemented in the program MEGA11 [48]. Web server iTOL [48, 49] (http://itol.embl.de) was used to visualize the phylogenetic tree and annotate the relative abundance of ASVs in each stage.

## Results

### Fluorescence in situ hybridization and transmission electron microscopy supported the horizontal transmission of microbes

**Figure 2:**
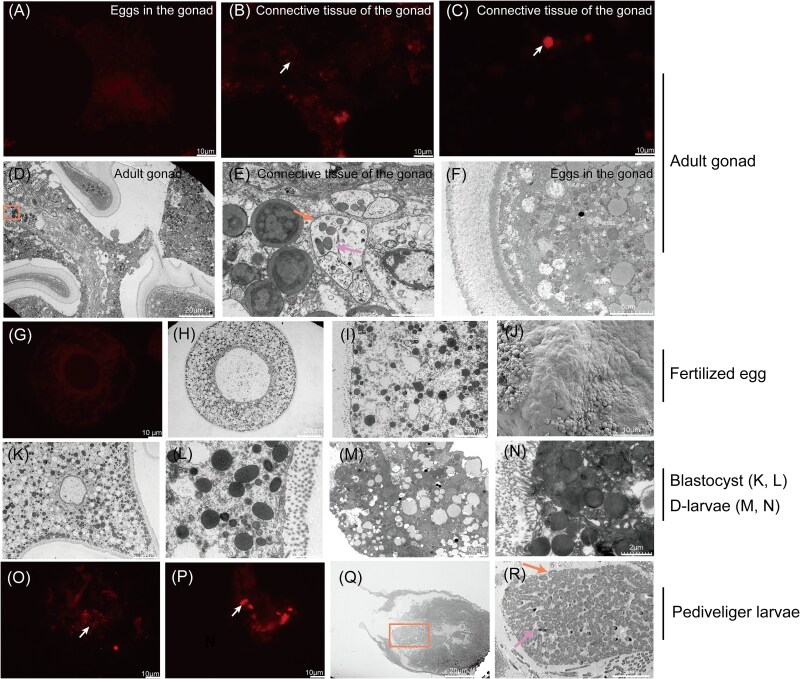
Microscopic observations of different stages of *Tridacna crocea*. Fluorescence microscope observation at different stages of *Tridacna crocea* (Cy3 labeled EUB338 probe [red], white arrows): (A) eggs in the gonad, (B) and (C) connective tissue of the gonad, (G) fertilized egg, (O) and (P) pediveliger larvae. Transmission electron microscopy: (D) adult gonad, the yellow box represents parts of the connective tissue of gonads containing symbiotic microbial structures, which is further enlarged in (E); (F) eggs in the adult gonad; (H) and (I) fertilized egg; (K) and (L) blastocyst; (M) and (N) D-larvae; (Q) pediveliger larvae, the yellow boxerepresents the symbiotic bacterial structure near the digestive gland of pediveliger larvae, which is further enlarged in (R). The yellow arrows represent the membrane structure, and the pink arrows represent symbiotic bacterial structures. Scanning electron microscopy: (J) fertilized eggs. Scale bars: (D), (H), and (Q): 20 μm; (A), (B), (C), (G), (J), (K), (M), (O), and (P): 10 μm; (F), (I), and (R): 5 μm; and (E), (L), and (N): 2 μm.

The adult gonad (TC0) and early developmental processes are depicted in Supplementary Fig. S1. Abundant eggs were visible in the adult gonad (TC0), characterized by polygonal cells and well-developed connective tissue. Following fertilization, the spherical eggs develop into multicellular embryos. As they grew into D-larvae (TC3), particular host organs emerged. During the development of the pediveliger larvae (TC4), host organs became more sophisticated (Supplementary Fig. S1). Both FISH and TEM analyses showed an absence of microbial structures within the gametes of gonads (Fig. 2A and F and Supplementary Figs S2B and S3) and within fertilized eggs (TC1) (Fig. 2G and I and Supplementary Fig. S2C), while bacterial aggregates were observed in the connective tissue of gonads (TC0) (Fig. 2C and E) and near the digestive gland of pediveliger larvae (TC4) with a highly visible membrane encasing (Fig. 2P and R). TEM further revealed the absence of microbial structures in both the blastocyst (TC2) (Fig. 2L) and the D-larvae (TC3) (Fig. 2N). Moreover, the SEM results revealed that the fertilized eggs (TC1) were spherical in shape with a rough surface texture, exhibiting irregularities and unevenness (Fig. 2J). Additionally, SEM images of eggs immediately after spawning clearly displayed microvilli structures on the egg surface (Supplementary Fig. S4C, F, and H), with microbial structures visible on the microvilli surface in Supplementary Fig. S4F.

### Diversity, ecological processes, and traceability analysis of microbial communities associated with early life stages of *Tridacna crocea*

**Figure 3:**
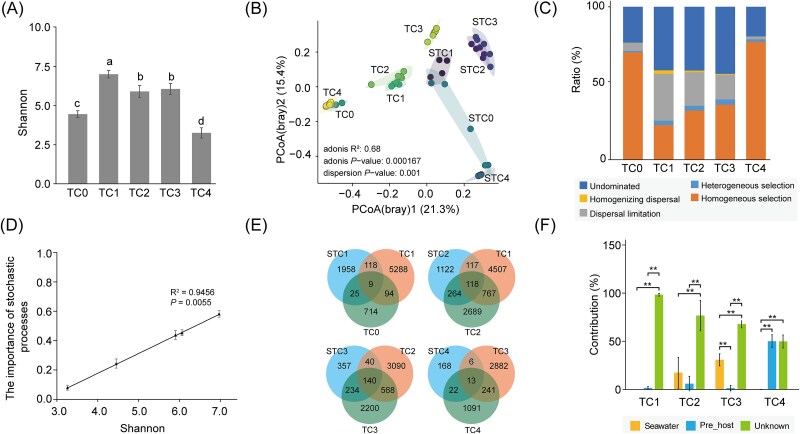
Bacterial communities associated with the selection pressure at different life stages. (A) Boxplot representing the Shannon α-diversity of bacterial communities. (B) PCoA using the Bray–Curtis dissimilarity index revealed the bacterial community structure. (C) Community assembly processes of bacterial communities. (D) Correlation analysis of the contribution of stochastic processes with α-diversity (Shannon) of bacterial communities. (E) Venn diagram analysis demonstrating the number of ASVs that were unique or shared in *Tridacna crocea* at each development stage. At the current time point, the host shares common bacterial ASVs with the host from the previous stage and the corresponding period of seawater. (F) The source model of *Tridacna crocea*’s microbiome was estimated using SourceTracker at different life stages. ^*^^*^P < .01 (*t*-test). Each sampling stage was identified as a sink, starting with the fertilized egg, and seawater samples from the current time point and hosts from the previous period were treated as potential sources. TC0: adult gonad, TC1: fertilized egg, TC2: blastocyst, TC3: D-larvae, TC4: pediveliger larvae. The “S” added before the biological sample represents the seawater sample of the corresponding stage.

The number of sequences in each sample was rarefied to 12 405 resulting in a rarefied ASV table. The saturation of the Shannon index dilution curve indicated that sufficient sequencing depth was achieved, as evidenced in Supplementary Fig. S5. As development progressed in the early stages, there was a gradual decrease in the bacterial Shannon-diversity index. Notably, the Shannon diversity significantly declined in pediveliger larvae (TC4) compared to embryo and D-larvae (TC3) stages (*P* < .05), although no significant difference was observed between blastocyst (TC2) and D-larvae (TC3) stages. In the adult gonads (TC0) of *T. crocea*, the Shannon-diversity index for bacteria was significantly lower than in embryo and D-larvae (TC3) stages (*P* < .05), but significantly higher than that in the pediveliger larvae (TC4) (*P* < .05) (Fig. 3A).

The PCoA revealed an obvious separation of the bacterial communities between *T. crocea*’s early life stages and seawater (Fig. 3B). Bacterial communities in the fertilized eggs (TC1) stage clustered closely with those in the blastocyst (TC2), yet showed a notable shift at the D-larvae (TC3) stage, while the pediveliger larvae (TC4) bacterial communities clustered more closely with those of the adult gonads (TC0) (Fig. 3B). Adonis analysis confirmed statistically significant differences in bacterial compositions across different developmental stages of *T. crocea* and seawater (*P* < .05) (Supplementary Table S1). PERMANOVA analysis further revealed significant effects of habitat (host vs water), time, and their interaction on bacterial community composition (permutations: 999, Fhabitat = 3.9367, *P* = .001; Ftime = 6.4050, *P* = .001; Fhabitat × time = 4.9305, *P* = .001). Notably, developmental time exerted the most significant influence on bacterial composition across the different stages of *T. crocea* (Supplementary Table S2).

The ecological model [50] was used to investigate the internal driving forces for all of the life stages of particular bacterial community types in *T. crocea*. Early developmental stages, including fertilized eggs (TC1), blastocyst (TC2), and D-larvae (TC3), were predominantly influenced by stochastic processes, particularly undominated processes and DL. These results suggested that microbial colonization at these stages was largely driven by random environmental factors, such as the availability of microbes in the surrounding seawater, rather than by host-specific selection. As development progressed, a marked transition toward deterministic processes was observed. This trend was most pronounced in the pediveliger larvae (TC4) and adult gonads (TC0), which exhibited high contributions from HoS. These findings indicated that at these stages, the host exerted strong selective pressures on its microbial communities, likely through the development of specialized structures or microenvironments that favored specific bacterial taxa (Fig. 3C). Moreover, a significantly positive correlation was identified between the relative importance of stochastic processes and bacterial Shannon diversity indices (*P* < .05) (Fig. 3D).

The Venn diagram analysis demonstrated that bacterial communities at each developmental stage shared ASVs with both the host’s bacterial communities from the previous stage and the corresponding period seawater bacterial communities (Fig. 3E). In the fertilized eggs (TC1), the shared ASVs predominantly affiliated with the family *Rhodobacteraceae* (Supplementary Fig. S6A). Bacterial unique ASVs accounted for >50% of the total ASVs across the whole development stages (Fig. 3E). SourceTracker analysis revealed that bacterial communities from prior biological stages increasingly contributed to the microbiota composition, reaching up to 50.10% in pediveliger larvae (TC4). In contrast, seawater contributed minor sources (<1% in TC1-fertilized eggs and TC4-pediveliger larvae, and < 40% in TC2-blastocyst and TC3-D-larvae), whereas unknown sources contributed significantly to the bacterial assemblage across developmental stages (Fig. 3F).

### Dynamics of the bacterial composition of the early life stages of *Tridacna crocea*

Bacterial communities within *T. crocea* adult gonads (TC0) were drastically different from those in subsequent early life stages. Initially, the family *Rhodobacteraceae* dominates in fertilized eggs (TC1), blastocyst (TC2), and D-larvae (TC3), with a notable shift toward a predominance of family *Endozoicomonadaceae* in the pediveliger larvae (TC4) and adult gonads (TC0), where this family accounts for over 50% of the community. Minor fluctuations are observed in the representation of certain bacterial groups throughout the early life stages. For example, the proportion of the family *Alteromonadaceae* gradually increased from the fertilized eggs (TC1) to the D-larvae (TC3) but diminished abruptly in the pediveliger larvae (TC4). The relative abundances of the families *Flavobacteriaceae*, *Staphylococcaceae*, *Corynebacteriaceae*, and *Micrococcaceae* increased from fertilized eggs (TC1) to blastocyst (TC2) and then decreased from D-larvae (TC3) to pediveliger larvae (TC4), while the family *Pirellulaceae* gradually decreased overall host’s early development (Supplementary Fig. S7A). Comparatively, seawater bacterial communities differed significantly from those associated with the host, with the family *Rhodobacteraceae* consistently showing high abundance across different developmental stages in seawater (Supplementary Fig. S7B).

**Figure 4:**
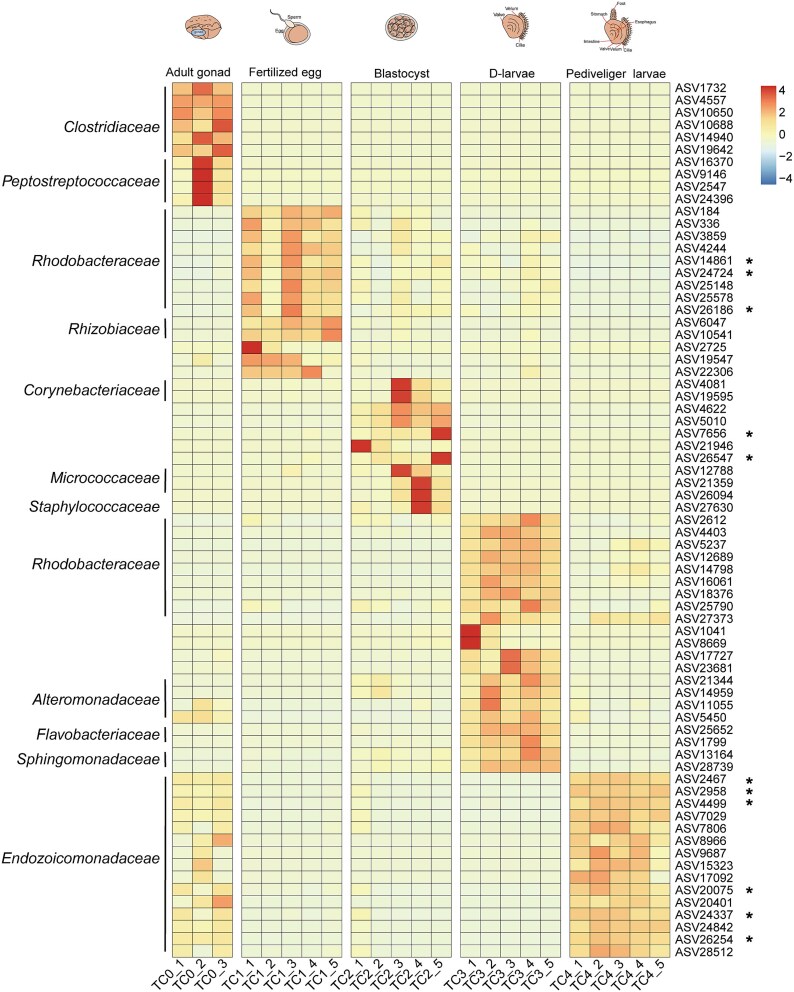
Bacterial ASVs that were identified as indicators at different life stages. LDA (linear discriminant analysis) > 3.5, the relative abundance of ASVs ≥0.01%. Regarding the scale from −4 to 4, this represents the range of values after the data has been standardized. The asterisks (^*^) denote ASVs that are shared across all developmental stages. TC0: adult gonad, TC1: fertilized egg, TC2: blastocyst, TC3: D-larvae, TC4: pediveliger larvae.

LEfSe analysis was used to identify biomarker ASVs at different development stages, with LDA scores (>3.5) revealing 71 biomarkers of significant relative abundance (Fig. 4; Supplementary Table S3). The significantly enriched ASVs were primarily affiliated with families such as *Clostridiaceae* and *Peptostreptococcaceae* in the adult gonads (TC0), *Rhodobacteraceae* and *Rhizobiaceae* in the fertilized eggs (TC1), *Corynebacteriaceae*, *Micrococcaceae*, and *Staphylococcaceae* in the blastocyst (TC2), *Flavobacteriaceae*, *Rhodobacteraceae*, *Alteromonadaceae*, and *Sphingomonadaceae* in the D-larvae (TC3), and *Endozoicomonadaceae* in the pediveliger larvae (TC4) (Fig. 4). ASVs that were initially absent in adult gonads (TC0) and fertilized eggs (TC1) gradually emerged as the host developed, in both seawater and biological samples, suggesting the acquisition of bacteria from seawater during the growth phase (Supplementary Fig. S8).

A total of 37 ASVs were found to be shared among the early life stages (Supplementary Fig. S9). Sixteen ASVs, including six *Endozoicomonadaceae* ASVs and ten *Rhodobacteraceae* ASVs, persisted across adult gonads (TC0), fertilized eggs (TC1), blastocyst (TC2), D-larvae (TC3), and pediveliger larvae (TC4) (Supplementary Figs S9 and S6C). The relative abundance of the six shared *Endozoicomonadaceae* ASVs remained stable from fertilized egg (TC1) to D-larvae (TC3), then gradually increased from D-larvae (TC3) to pediveliger larvae (TC4). In contrast, the seven shared *Rhodobacteraceae* ASVs were stable during the embryonic stage, increased from blastocyst (TC2) to D-larvae (TC3), but decreased in pediveliger larvae (TC4), with three ASVs showing a continuous decline from fertilized eggs (TC1) to pediveliger larvae (TC4) (Supplementary Fig. S6A and S6C). Most of these shared ASVs in all development stages exhibited significant differences (Fig. 4). Additionally, six *Rhodobacteraceae* ASVs were found to be shared among adult gonads (TC0), fertilized eggs (TC1), and the corresponding seawater of fertilized eggs (STC1), intersecting with ASVs shared across all stages of host development (Supplementary Fig. S6A).

### Co-occurrence networks revealed more positive associations among microbes across different life stages of *Tridacna crocea*

After filtering out low-abundance ASVs, the nodes in the bacterial community networks consistently maintained 150 across different developmental stages. The topological properties of these networks revealed distinct co-occurrence patterns among bacterial communities at different developmental stages (Supplementary Table S4). Notably, the edge number (1921), average degree (25.61), and mean clustering coefficient (1) of the network in the adult gonads (TC0) microbial community were much larger than the other early stages. This pattern followed an N-shaped trajectory across development, indicating an initial increase, followed by a decrease, and then another increase in complexity as the host developed. In the adult gonads (TC0) microbial community, the shorter diameters (1) and average path length (1) compared to those in the early life stage suggested a higher efficiency of information transfer among bacteria, which enabled quicker communication and resource sharing within the community. This enhanced efficiency likely facilitated improved overall function and stability of the microbiome as the host matured. A higher modularity will increase the stability of the network [51], as the host continued to mature, the modularity of the bacterial network structure gradually increased, suggesting that bacterial networks were becoming more and more stable (Supplementary Table S4).

As the host developed, the composition and interactions of bacterial communities shifted. For example, the phyla Proteobacteria and Firmicutes dominated in the adult gonads (TC0), while phyla Proteobacteria and Cyanobacteria dominated in the blastocyst (TC2) stage. In the fertilized eggs (TC1), D-larvae (TC3), and pediveliger larvae (TC4), the phyla Proteobacteria and Bacteroidota predominated. Positive associations among ASVs were more prevalent than negative associations across different stages of host development (Supplementary Fig. S10). Notably, 15 keystone ASVs were identified across early life stages, but there were no common keystone ASVs shared among these stages, suggesting that different stage-specific keystone ASVs are critical at each developmental stage. The number of keystone ASVs in the bacterial interaction networks was higher in fertilized eggs (TC1) (six ASVs) and D-larvae (TC3) (seven ASVs) compared to the blastocyst (TC2) (one ASV) and pediveliger larvae (TC4) (one ASV). However, due to limited sample replication, no keystone ASVs were identified in the adult gonads (TC0) (Supplementary Fig. S11).

### Diversity and phylogenetic analysis of the genus *Endozoicomonas*

**Figure 5:**
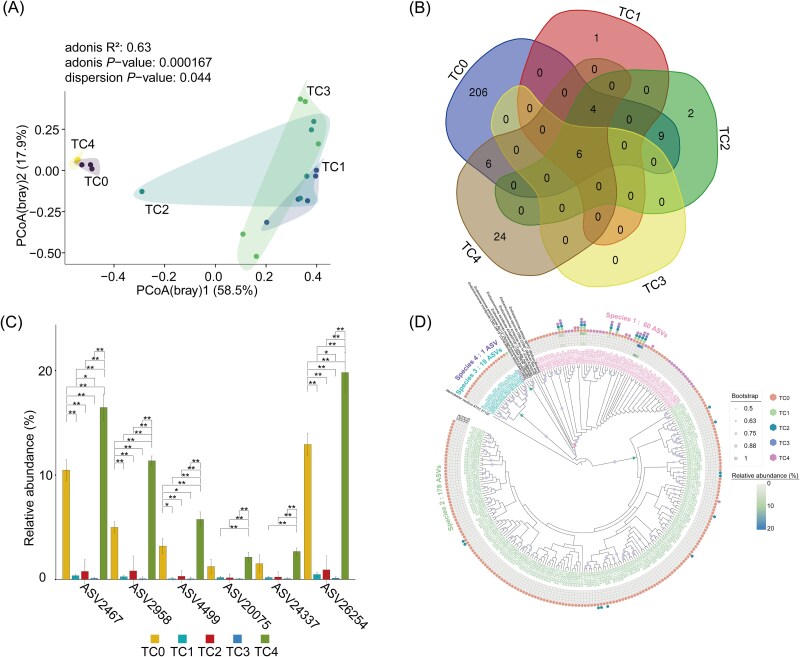
Diversity and phylogenetic affinities of the genus *Endozoicomonas*. (A) PCoA of genus *Endozoicomonas* at ASVs level in different developmental stages*.* (B) Venn diagrams showing the number of the genus *Endozoicomonas* ASVs that were unique or shared in different developmental stages of *Tridacna crocea*. (C) The relative abundance of six shared *Endozoicomonas* ASVs in each developmental stage. ^*^P < .05, ^*^^*^P < .01 (*t*-test). (D) NJ phylogenetic tree among genus *Endozoicomonas* ASVs from *Tridacna crocea* and reference *Endozoicomonas* strains from other marine invertebrates (bivalve, coral, Ascidiaceae, and sponges, reference sequences were downloaded from the EzBioCloud database). The relative abundance of ASVs in each development stage is visualized by a heatmap. Bootstrap values of >50% are shown next to branch nodes. The green-pointed stars indicate that ASVs are mainly derived from the gonads, and the pink-pointed star indicates that ASVs are mainly derived from the gonads and the early developmental stages. The different colored hexagons represent ASVs from different development stages. Different colors of ASVs represent different *Endozoicomonas* species (threshold 97%). TC0: adult gonad, TC1: fertilized egg, TC2: blastocyst, TC3: D-larvae, TC4: pediveliger larvae.

In this study, a high proportion of genus *Endozoicomonas* was found in adult gonads (TC0) and pediveliger larvae (TC4). There was no significant difference observed in the relative abundance of the genus *Endozoicomonas* between the adult gonads (TC0) (59.54%) and pediveliger larvae (TC4) (80.86%). However, both stages exhibited significantly higher relative abundances compared to fertilized eggs (TC1), blastocyst (TC2), and D-larvae (TC3) stages (0.54%–4.56%, *P* < .05) (Supplementary Fig. S12). The PCoA results indicated significant differences in the composition of the genus *Endozoicomonas* communities among different developmental stages (*P* < .05) (Fig. 5A). The Adonis analysis confirmed significant differences in the genus *Endozoicomonas* composition between adult gonads (TC0) and pediveliger larvae (TC4), as well as when comparing both to other early developmental stages (*P* < .05). However, there were no significant differences among fertilized eggs (TC1), blastocyst (TC2), and D-larvae (TC3) stages in the composition of the genus *Endozoicomonas* (Supplementary Table S5). The annotated results indicated the presence of 258 ASVs belonging to the genus *Endozoicomonas*, with 6 ASVs consistently present across adult gonads (TC0) and all early developmental stages (Fig. 5B). A notable increase in the relative abundance of ASV2467, ASV2958, ASV4499, and ASV26254 was observed in pediveliger larvae (TC4) compared to other stages (*P* < .05) (Fig. 5C). Additionally, unique ASVs were identified in fertilized eggs (TC1) (one ASV), blastocyst (TC2) (two ASVs), pediveliger larvae (TC4) (24 ASVs), and adult gonads (TC0) (206 ASVs), whereas no unique ASVs were detected in D-larvae (TC3) (Fig. 5B; Supplementary Table S6).

*Endozoicomonas* ASVs associated with *T. crocea* exhibited a high degree of host specificity, forming distinct clusters separate from other *Endozoicomonas* species associated with various invertebrates (Fig. 5D). ASVs can represent biological variance among microbial strains within the same species. The clustering of ASVs with 16S rRNA gene similarity of 97% or greater is used as a threshold for species-level classification [52]. Using a local blast service with an all-versus-all strategy, we observed a high diversity and divergence of the *Endozoicomonas* strains (ASVs) across different developmental stages (Supplementary Table S7). Notably, 60 out of the 258 ASVs were identified as a known *Endozoicomonas* species, showing the highest 16S rRNA gene similarity (99.86%) to *Endozoicomonas atrinae* WP70^T^, which was isolated from the gut of a comb pen shell (*Atrina pectinata*) collected from the southern sea of Yeosu in Korea [53]. The remaining 198 ASVs showed <97% similarity to the known *Endozoicomonas* species, suggesting they represent novel species. The phylogenetic analysis further demonstrated that they were divided into four distinct clades, representing four species (Fig. 5D). The top10 *Endozoicomonas* ASVs across all life stages (with relative abundances ranging from 0.25% to 6.33%), predominantly identified in adult gonads (TC0) and pediveliger larvae (TC4), clustered in the same clade affiliated to the same Species 1. Conversely, the *Endozoicomonas* ASVs, which were mostly from the adult gonads (TC0) and had relative abundances of <0.1% throughout all life stages, were grouped into three different clades that correspond to three new *Endozoicomonas* species. Furthermore, the greatest diversity of *Endozoicomonas* species and strains was found in adult gonads (TC0), with 36 strains affiliated to Species 1, 19 strains to Species 3, and 176 strains to Species 2. This was followed by pediveliger larvae (TC4), which had 39 strains affiliated to Species 1 and 1 strain to Species 2; blastocyst (TC2), with 10 strains affiliated to Species 1 and 11 strains to Species 2; fertilized eggs (TC1), containing 10 strains affiliated to Species 1 and 1 strain to Species 4; and D-larvae (TC3), with 6 strains affiliated to Species 1 (Fig. 5D).

## Discussion

### Bacteria are transmitted horizontally to the tissues of the *Tridacna crocea*

**Figure 6:**
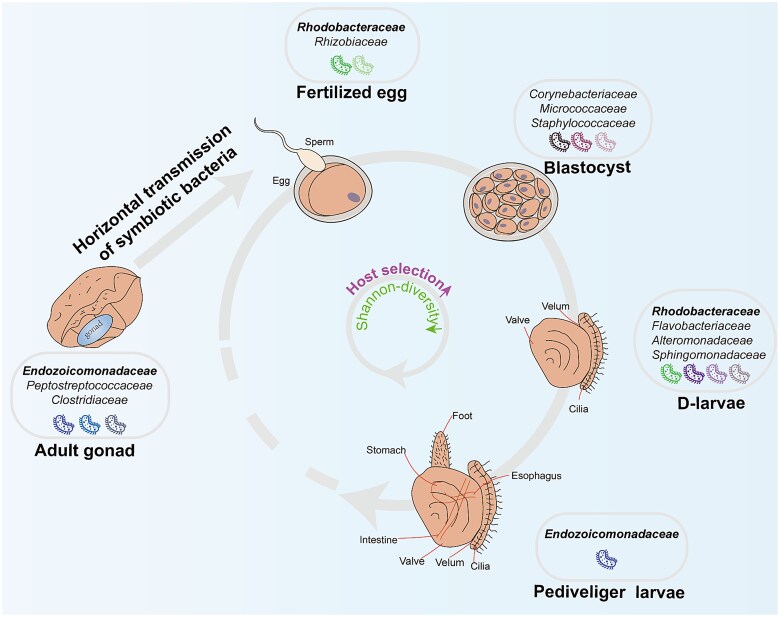
Conceptual map of horizontal transmission of symbiotic microorganisms and microbial community dynamics in the early stages of *Tridacna crocea.* The different colors of bacteria represent different microbial taxa.

The integration of microbial symbionts into the reproductive and developmental processes of their hosts can occur through various modes of transmission, including horizontal, vertical, and mixed pathways [14]. In this study, microscopic observations (TEM or FISH) revealed the absence of symbiotic bacteria in the gametes of gonads, fertilized eggs, blastocyst, and D-larvae. However, symbiotic bacterial signals and aggregates were detected in the connective tissue of the gonads and near the digestive gland of pediveliger larvae. SEM further showed that the surface of freshly released eggs was covered with microvilli, which could carry microorganisms. While 16S rRNA sequencing data demonstrated the existence of bacteria across different life stages, including fertilized eggs, the combined evidence from SEM, TEM, and FISH analyses suggested that the bacteria detected through 16S rRNA sequencing in fertilized eggs were likely attached to the egg’s microvilli surface rather than being internalized within the host. These findings collectively point to a potential for surface-mediated bacterial transport (Fig. 6). The specific bacterial transmission mechanism may involve two potential pathways. Firstly, adult *T. crocea* could facilitate the direct transfer of bacteria to gametes during spawning, promoting external vertical transmission rather than strict vertical inheritance. Secondly, bacteria could have been released into the water column during spawning, promoting horizontal transmission and thereby increasing the likelihood of bacterial acquisition by the offspring. This mechanism aligns with previous hypotheses in corals, suggesting that parental bacteria may influence offspring microbial communities during reproduction through this mode of horizontal transmission [54]. For example, ASV from the family *Endozoicomonadaceae*, typified by ASV26254, was consistently found in the gonad and all early life stages, with the highest relative abundance in pediveliger larvae, followed by the gonads. Intriguingly, ASV26254 displayed a higher relative abundance in seawater surrounding the gonads at the time of sampling compared to other stages, with no detection in seawater from other developmental stages, suggesting that the bacterial communities from adult *T. crocea* could enrich the water column and promote uptake by offspring in natural settings. Furthermore, shared ASVs were identified among adult gonads, fertilized eggs, and the corresponding seawater phase, with some overlap with ASVs shared between the gonads and all early life stages. Most of these shared ASVs between gonads and early life stages exhibited significant and increasing trends, indicating their essential role. The presence of unique bacterial ASVs at each developmental stage likely resulted from selective acquisition from the environment. Given the enrichment of the family *Rhodobacteraceae* in early host stages and its prevalence in seawater, it is postulated that this family predominantly enters the host through horizontal transmission from the surrounding seawater. Research on bivalves has shown that different hosts have different transmission modes of symbiotic bacteria. For instance, *Solemya reidi* [55], *Calyptogena soyoae* [56], and *Isorropodon bigoti* [18] exhibit vertical transmission of symbiotic bacteria from parents to descendants via the female gametes, whereas *Loripes lacteus* [19] and *Conchocele bisecta* [20] demonstrate the horizontal transmission of symbiotic bacteria. These different transmission strategies can reflect the complexity of interactions between hosts and microbes. Additionally, each transmission strategy comes with its own set of costs, microbial communities transmitted vertically tend to be more stable and play a symbiotic role [14], whereas those acquired horizontally depend on the distribution of environmental microbes and conditions [14]. The mechanism of bacterial acquisition during spawning in *T. crocea* remains unclear, potentially involving two primary modes: (i) external vertical transmission through bacterial adherence to egg surface microvilli and (ii) horizontal transmission via bacteria released into the water column by spawning adults or/and colonization by ambient seawater microbiota. Based on the above discussions and the absence of internalized bacteria in fertilized eggs or adult gonadal gametes, we prefer to classify the symbiotic bacteria acquisition in *T. crocea* as horizontal transmission. However, we acknowledge the potential role of external vertical transmission in facilitating microbial adherence via egg surface microvilli and emphasize the need for further investigation to elucidate the precise mechanisms underlying bacterial acquisition and colonization.

### Ecological deterministic processes and host selection effects potentially shape microbiome assembly in early developmental stages

It is well known that hosts can acquire symbiotic microorganisms from their surrounding seawater environment, and this dynamic process persists throughout the entire life cycle of organisms. In our study, the bacterial community of *T. crocea* was notably distinct from that of the seawater community, despite a significant number of ASVs being shared between the two communities. SourceTracker analysis indicated that only a small fraction of the bacterial community in *T. crocea* originated from the previous developmental stage (internal source) and seawater (external source) before the pediveliger larvae stage. It seemed unlikely that the microbiota of *T. crocea* in a cultivation setting would not be substantially influenced by the surrounding seawater microbiota, as external microorganisms for *T. crocea* must primarily originate from the seawater. The discrepancy might be attributed to the continuous and dynamic nature of microorganisms in the seawater environment [57], coupled with the ongoing development of the host. The biological samples we sequenced were collected during sampling processes, which may not have captured all microbial taxa interacting with the host across all time scales of seawater. Additionally, sequencing technology itself may have limitations, resulting in a notable proportion of “unknown” taxa. Interestingly, during the pediveliger larvae stage, there was a significant increase in the proportion of bacteria sourced from internal origins. This increase could be attributed to the initiation of feeding in D-shaped larvae being associated with the emergence of internal gut microbial communities [21], potentially resulting in a higher proportion of host microbiota originating from the previous stage in the pediveliger larvae stage.

During development, hosts not only acquire symbiotic bacteria through environmental dispersal but also exert a strong selective effect on shaping symbiotic communities [21, 58]. In our study, the null model analysis revealed that the bacterial community was initially assembled stochastically but underwent a significant transition to the deterministic assembly during the pediveliger larvae and gonads. Given that recruitment and enrichment processes are energetically costly for the host, extensive selection strategies may not be employed before the pediveliger larvae stage [14, 59]. During the embryonic stage, it is likely that the microbial community from seawater primarily adheres to the biosample surface randomly, thereby maintaining a higher diversity in our study. The higher α-diversity of predominantly adhered bacteria during the embryo stages potentially serves as a microbial reservoir for subsequent host selection processes by the host. Once symbiotic bacteria establish and settle into their final niches, the host typically intensifies the stringency of selection as it develops [14]. The early acquisition of microbial diversity may also be attributed to an immature immune system [60]. Reports suggest that host selection for microbial communities increases with the maturity of zebrafish [61]. In our study, a strong selective sweep led to a decrease in community α-diversity and a high proportion of internal microbes, particularly evident in pediveliger larvae, indicating a winnowing process as *T. crocea* matures. This winnowing process is thought to refine the microbiome to better match the host’s needs and environmental conditions [62]. Ongoing research aims to explore the potential role of the microbiome in the immune system development of *T. crocea*, aiming to refine and clarify these interactions.

Besides the increasing importance of deterministic processes with host maturation, the structure of bacterial communities in pediveliger larvae and gonads exhibited closer clustering compared to D-larvae and embryo stages, accompanied by more intricate co-network microbial interactions. Complex microbial networks have been shown to enhance stability in network structure [63], whereas simpler networks can propagate environmental changes rapidly across the entire network, potentially causing instability [64]. It indicated that the bacteria developed into a relatively mature community as the host matured, contributing to the establishment of a stable state. Notably, positive correlations predominated in bacterial interaction relationships across different developmental stages of *T. crocea*, indicating shared ecological niches or close symbiotic relationships [65]. These dynamics potentially facilitate bacterial colonization and stability during early developmental stages.

### Microbial dynamics and potential symbiotic relationships in *Tridacna crocea* development

Giant clams harbored significant microbial taxa across their different developmental stages. The shift in bacterial community biomarkers and keystone microbial taxa across different developmental stages was likely associated with changes in the host’s physiological status and microbial source. Studies on *C. sikamea* and bivalve filter feeding showed that microbial communities were shaped by both host development and environmental factors [21, 66]. Research on giant clams (Bivalvia: Cardiidae: *Tridacninae*) further suggested that these shifts were closely tied to host physiological changes [67]. During the fertilized egg and D-larvae stages, the predominant taxa belonged to the family *Rhodobacteraceae*. As discussed in the first section, this prevalence may be attributed to the random dispersal and adhesion of microbial communities from seawater to the biological samples, particularly when the host’s immune system is immature. Simultaneously, the family *Rhodobacteraceae* has also been shown to possess capabilities to perform photosynthesis in anaerobic environments [68], degrade hydrocarbon [69], synthesize vitamin B_12_ [70], produce antibacterial compound tropodithietic acid [71], and commonly colonize host organisms to promote host health [72–75]. Therefore, the family *Rhodobacteraceae* may play a positive role in promoting digestion, providing nutrients, and inhibiting pathogens. In the blastocyst stage, the enrichment of the families *Staphylococcaceae*, *Corynebacteriaceae*, and *Micrococcaceae* was observed. Previous literature has reported that families *Staphylococcaceae* [76], *Corynebacteriaceae* [77], and *Micrococcaceae* [78] are often potentially pathogenic bacteria. However, no apparent pathogenicity was observed based on their status in this context. Therefore, the roles of these taxa deserve further investigation.

ASVs affiliated with the genus *Endozoicomonas* were significantly enriched in pediveliger larvae. Although we did not conduct continuous sequencing from the pediveliger larvae through to the adult developmental stages, we hypothesize that the genus *Endozoicomonas* may be an important group during this intermediate process. This hypothesis was supported by its high relative abundance in adult gonads and similarities in community compositions between pediveliger larvae and adult gonads. Interestingly, phylogenetic tree analysis revealed the adult gonads harbored the highest number of unique strains (ASVs) and *Endozoicomonas*-related phylotypes, followed by the pediveliger larvae. The presence of six shared strains from *Endozoicomonas* species 1 across all developmental stages implied a potential influence on microbial community composition and host health. A high *Endozoicomonas* strain genetic diversity of *Endozoicomonas*-related phylotypes associated with *T. crocea* was revealed in this study. Specifically, the strains from adult gonads showed the highest genetic diversity of *Endozoicomonas-*related phylotypes compared to those from pediveliger larvae, both of which exceeded those from blastocyst, fertilized eggs, and D-larvae. This higher level of diversity of strain and phylotypes may contribute to increased competitive advantage during development. However, the top10 *Endozoicomonas* strains in all life stages (relative abundance 0.25%–6.33%), found in the gonad and pediveliger larvae belonged to the same species, indicating potential differences in their adaptation capabilities. Additionally, the *Endozoicomonas* strains found in *T. crocea* formed a distinct cluster separate from other *Endozoicomonas* species strains found in different invertebrates, suggesting a certain degree of conservation and host specialization, as well as complex patterns of host-symbiont species co-diversification, geographical adaptation [79]. We found a low relative abundance of the genus *Endozoicomonas* in seawater, with instances where it remained undetected during certain sampling stages. However, hosts may gradually accumulate genus *Endozoicomonas*, establishing a symbiotic relationship and forming higher *Endozoicomonas-*related phylotypes. Genus *Endozoicomonas* likely plays critical roles in host nutrient acquisition and cycling, polysaccharide degradation, dimethylsulfoniopropionate (DMSP) degradation, and contributes to the host’s health, acclimatization, and adaptation [47, 79–82]. Besides, TEM and FISH microscopy results revealed bacterial aggregates within the connective tissue of gonads and pediveliger larvae. Previous literature has reported that genus *Endozoicomonas* species typically reside in aggregates within host tissues [79, 80, 83, 84]. It is suggested that these bacterial aggregates in this study may be attributed to the highly abundant genus *Endozoicomonas*. Further microscopic observations revealed that the bacterial aggregates seemed to be surrounded by a membranous layer, which appeared to be disintegrating in pediveliger larvae. This observation suggested a complex and dynamic symbiotic relationship within the host’s tissues.

## Conclusions

In conclusion, this study sheds light on the transmission modes and dynamics of symbiotic bacteria during the early developmental stages of the giant clam *T. crocea*. Utilizing microscopic techniques, and given the absence of internalized bacteria in fertilized eggs or adult gonad gametes, we prefer to classify the acquisition of symbiotic bacteria as horizontal transmission. Our findings highlighted the complex interplay between host selective sweep and the assembly of the microbiome during early developmental stages. Furthermore, the presence of key microbial groups, including the family *Rhodobacteraceae* and genus *Endozoicomonas*, suggested their potential contributions to host health, nutrient acquisition, and adaptation. The genus *Endozoicomonas*, in particular, exhibited relatively high phylotype diversity and host specialization. Overall, this study provides valuable insights into the establishment of symbiosis between giant clams and microorganisms.

## Supplementary Material

Supplementary_figures_ycaf037

Supplementary_tables_ycaf037

Supplementary_methods_ycaf037

Script_ycaf037

## Data Availability

The 16S rRNA raw data are publicly available in the NCBI Sequence Read Archive under BioProject accession number PRJNA1126069.
