# Supplementary material for: Horizontal transmission of symbiotic bacteria and host selective sweep in the giant clam Tridacna crocea
Source: ISME Commun. 2025 Mar 2;5(1):ycaf037. doi: 10.1093/ismeco/ycaf037 (PMC11919647; doi:10.1093/ismeco/ycaf037)
Supplement: Supplementary_figures_ycaf037 [file supplementary_figures_ycaf037.pdf]

Horizontal transmission of symbiotic bacteria and host selective sweep in the giant clam *Tridacna crocea*

Cong Liu [1 2 #], Jian Zhang [1 #], Qiqi Li [1], Yuehuan Zhang [1], Si Zhang [1 3], Ziniu Yu [1], Jun Li [1 3*], Jie Li [1 3*]

[1] CAS Key Laboratory of Tropical Marine Bio-resources and Ecology, South China Sea Institute of Oceanology, Chinese Academy of Sciences, Guangzhou 510301, China
[2] University of Chinese Academy of Sciences, Beijing 100049, China
[3] Sanya National Marine Ecosystem Research Station, Chinese Academy of Sciences, Sanya 572000, China

*Corresponding authors. Jie Li, CAS Key Laboratory of Tropical Marine Bio-resources and Ecology, South China Sea Institute of Oceanology, Chinese Academy of Sciences, Guangzhou 510301, China. Email: lijietaren@scsio.ac.cn; Jun Li, CAS Key Laboratory of Tropical Marine Bio-resources and Ecology, South China Sea Institute of Oceanology, Chinese Academy of Sciences, Guangzhou 510301, China. Email: jun.li@scsio.ac.cn.
[#]Cong Liu and Jian Zhang contributed equally to this study.

Supplementary Figures

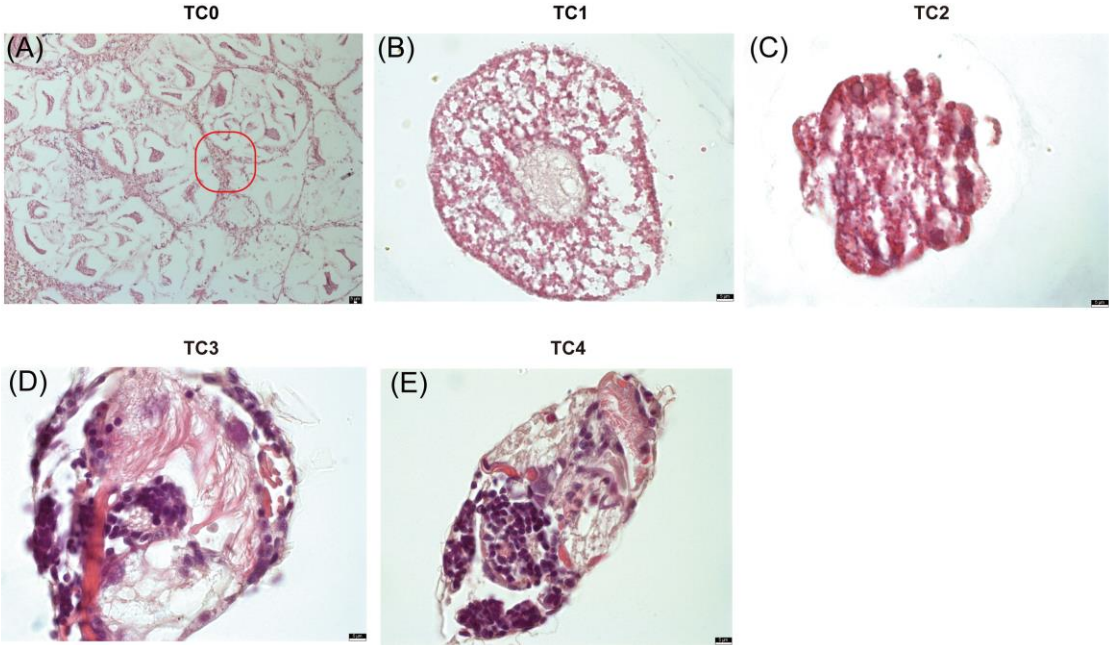

Figure S1. Hematoxylin–eosin staining. (A) Adult gonad, (B) Fertilized egg, (C) Blastocyst, (D) D-larvae, (E) Pediveliger larvae. The red box indicates the connective tissue sites of the gonad. TC0: adult gonad, TC1: fertilized egg, TC2: blastocyst, TC3: D-larvae, TC4: pediveliger larvae. Scale bars, 5 μm.

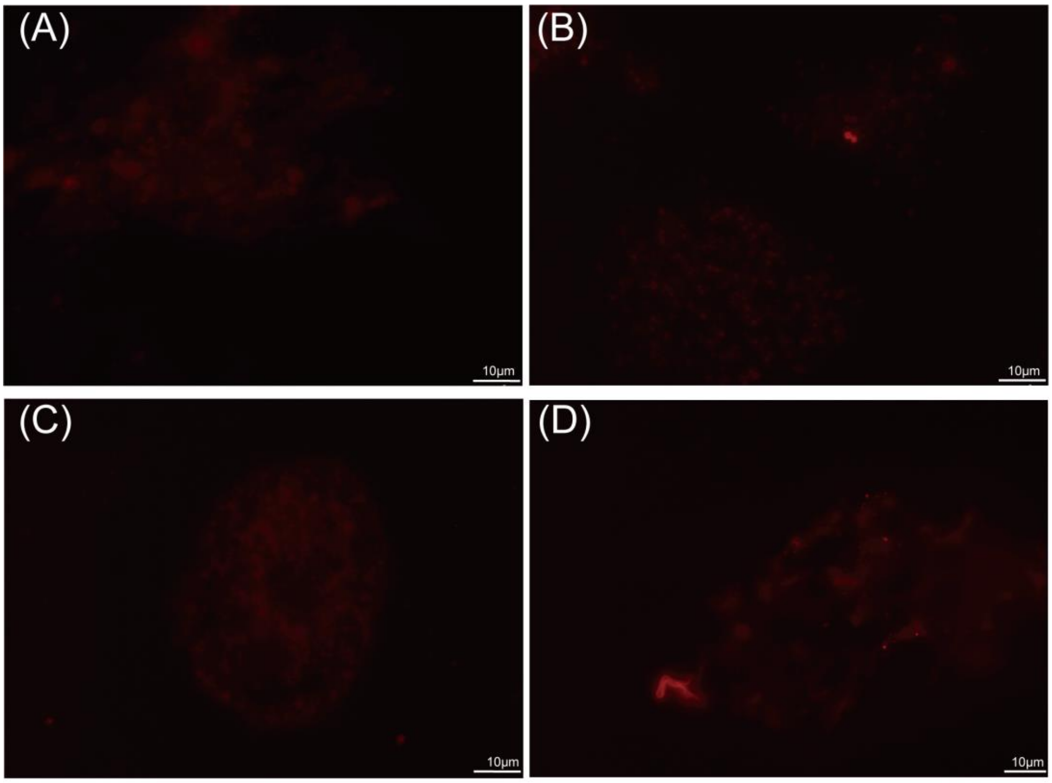

Figure S2. Fluorescence microscope observation (Cy3 labeled NONEUB338 probe [red]). (A) Connective tissue of the gonad, (B) Egg in the gonad, (C) Fertilized egg, (D) Pediveliger larvae. Scale bars, 10 μm.

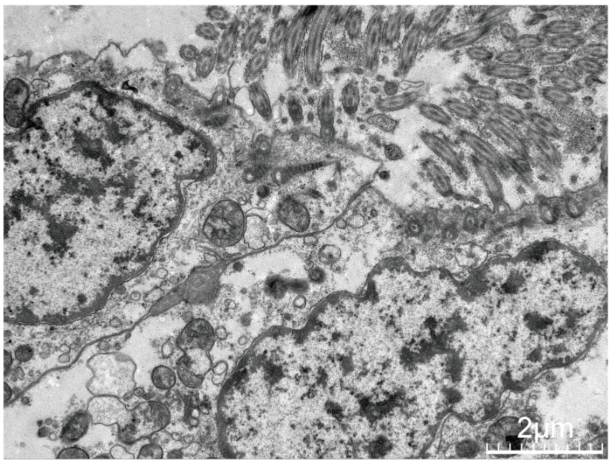

Figure S3. Transmission electron microscopy (TEM) image of sperm is shown. Scale bars, 2 μm.

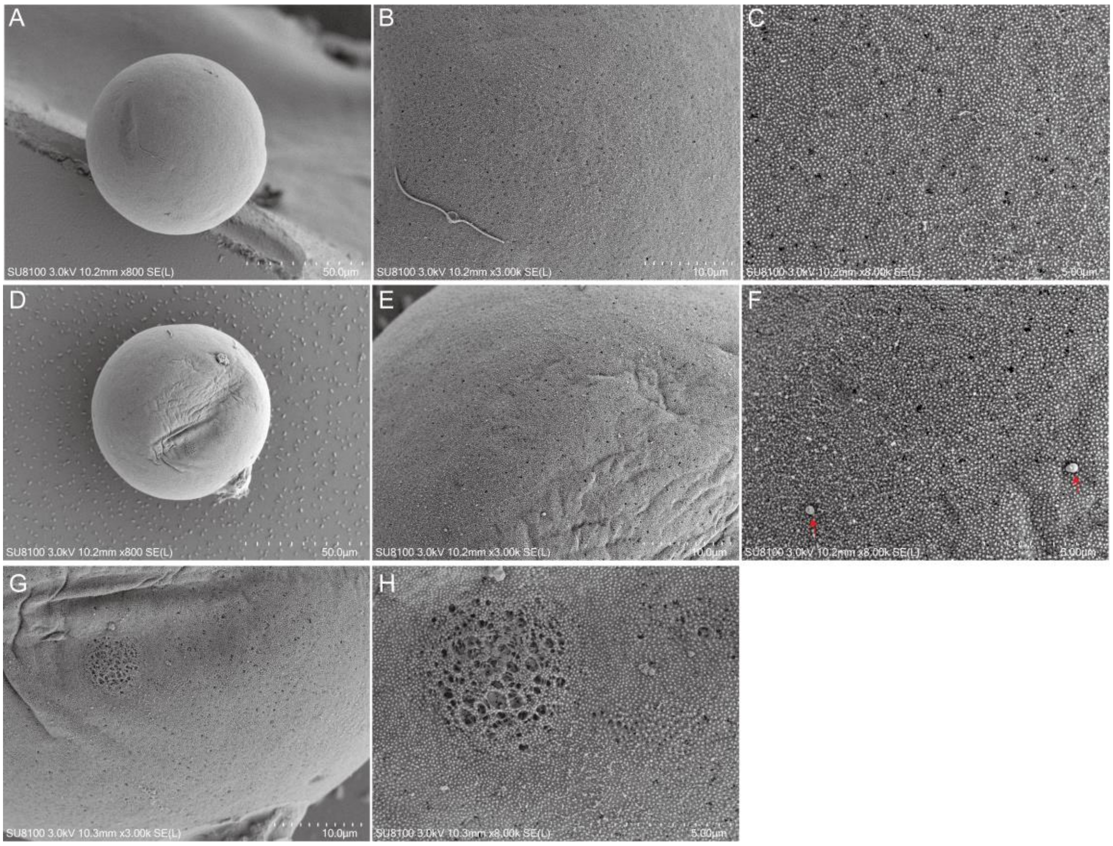

Figure S4. Scanning electron microscopy. (A) Egg of *Tridacna crocea* immediately after release, further enlarged in (B), and (C). (D) Egg of *Tridacna crocea* immediately after release, from a different individual, further enlarged in (E), (F), (G), and (H). The red arrow in (F) indicates bacterial structures carried on the microvilli of the egg surface. Scale bars: (A), and (D): 50 μm, (B), (E), and (G): 10 μm, (C), (F), and (H): 5 μm.

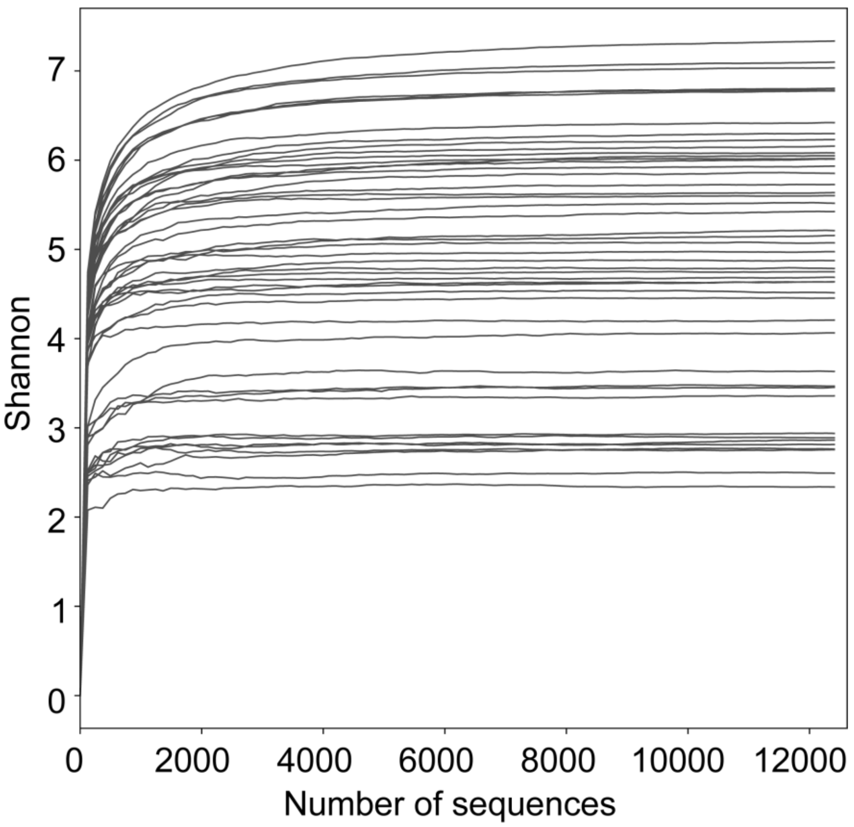

Figure S5. Shannon index dilution curve.

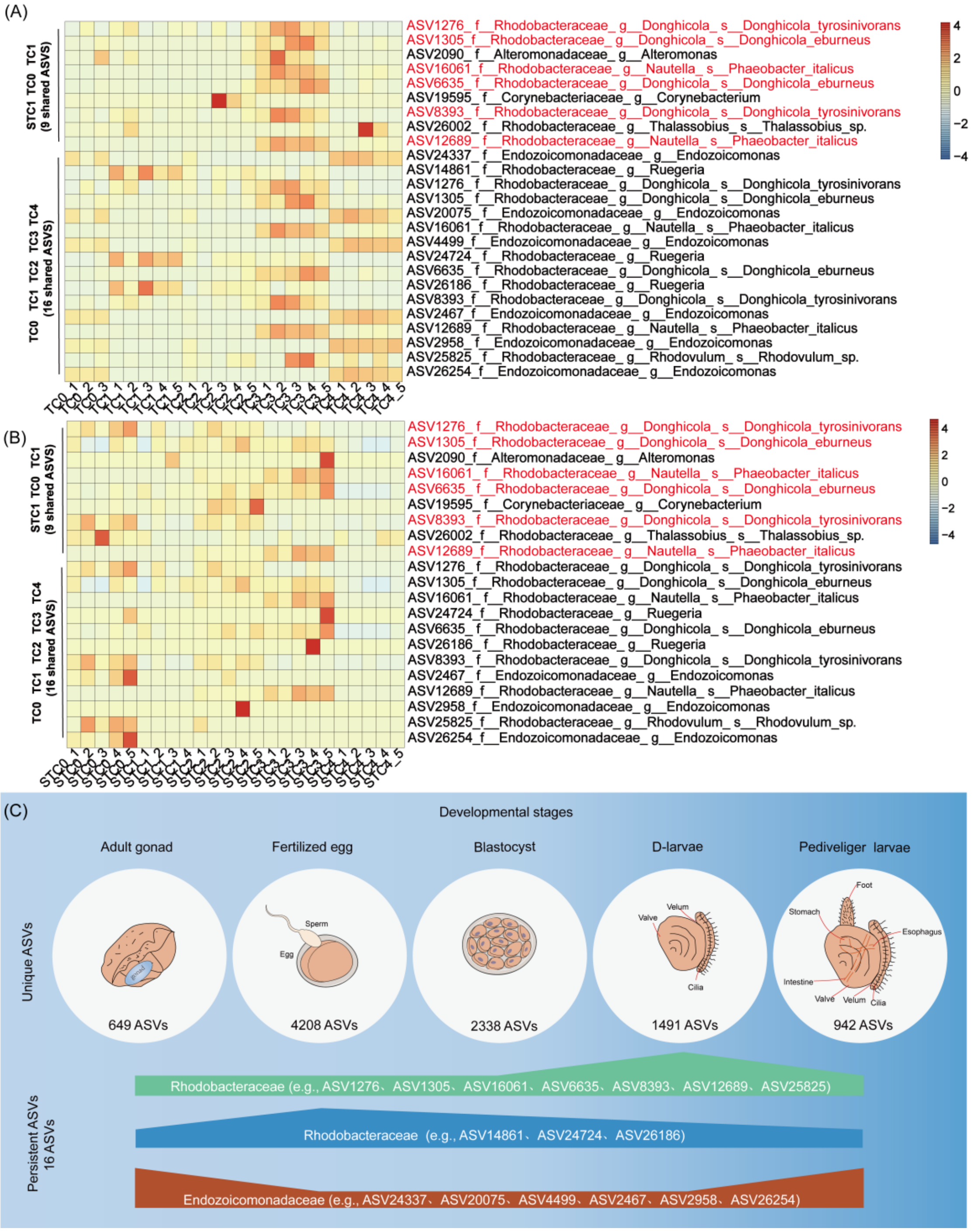

Figure S6. The dynamic changes of microbial communities. The relative abundance of the ASVs that were shared in *Tridacna crocea* (A) and seawater (B) at different development stages. Regarding the scale from -4 to 4, this represents the range of values after the data has been standardized. (C) Summary of dynamic changes of unique and shared ASVs. TC0: adult gonad, TC1: fertilized egg, TC2: blastocyst, TC3: D-larvae, TC4: pediveliger larvae. The "S" added before the biological sample represents the seawater sample of the corresponding stage. The red font represents the overlapping ASVs.

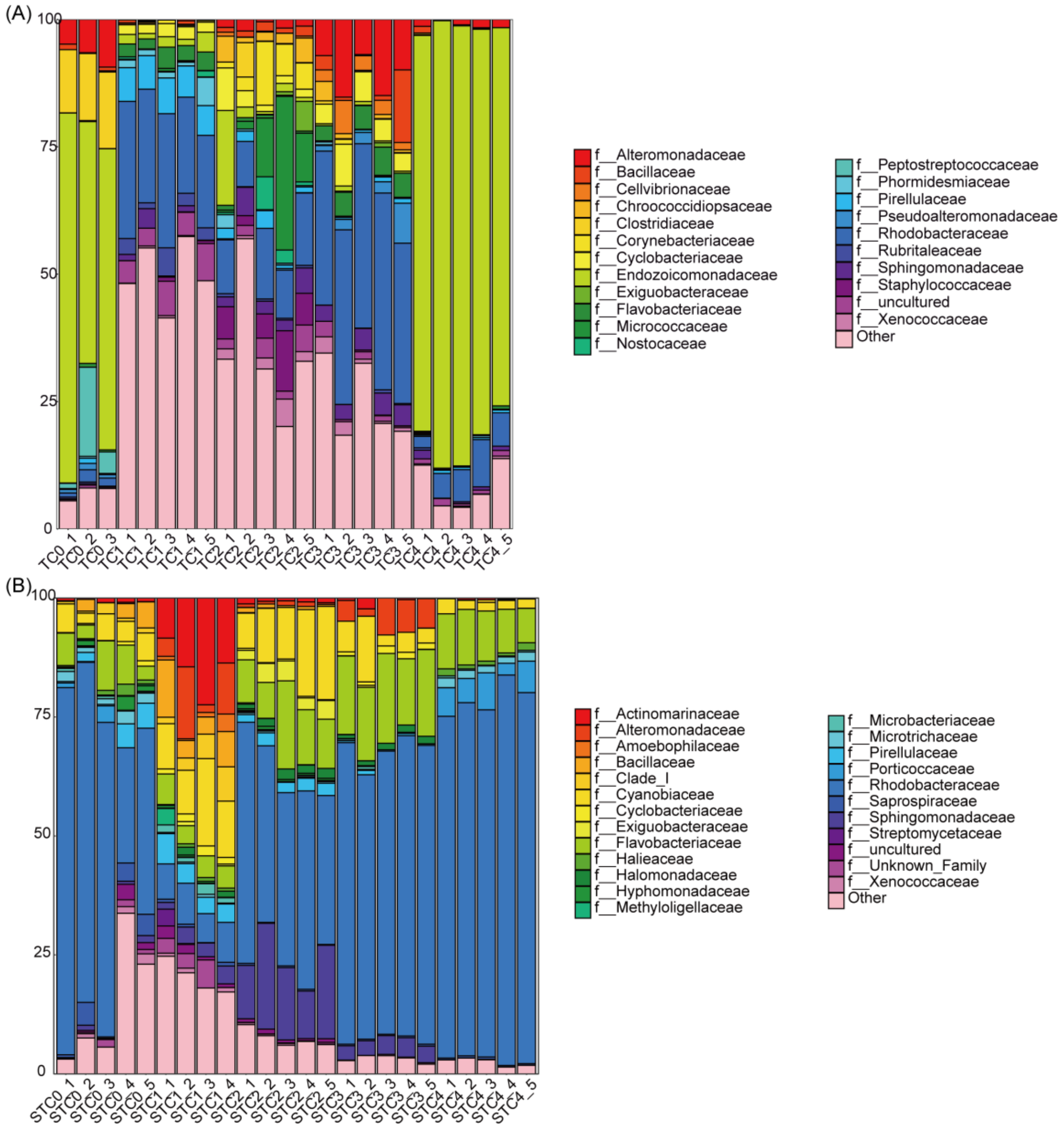

Figure S7. Analysis of bacterial composition at the family level. (A) Composition analysis at the family level of bacteria in the host, "other" means that the relative abundance of bacteria is less than 5%. (B) Composition analysis at the family level of bacteria in seawater, "other" means that the relative abundance of bacteria is less than 2%. TC0: adult gonad, TC1: fertilized egg, TC2: blastocyst, TC3: D-larvae, TC4: pediveliger larvae. The "S" added before the biological sample represents the seawater sample of the corresponding stage.

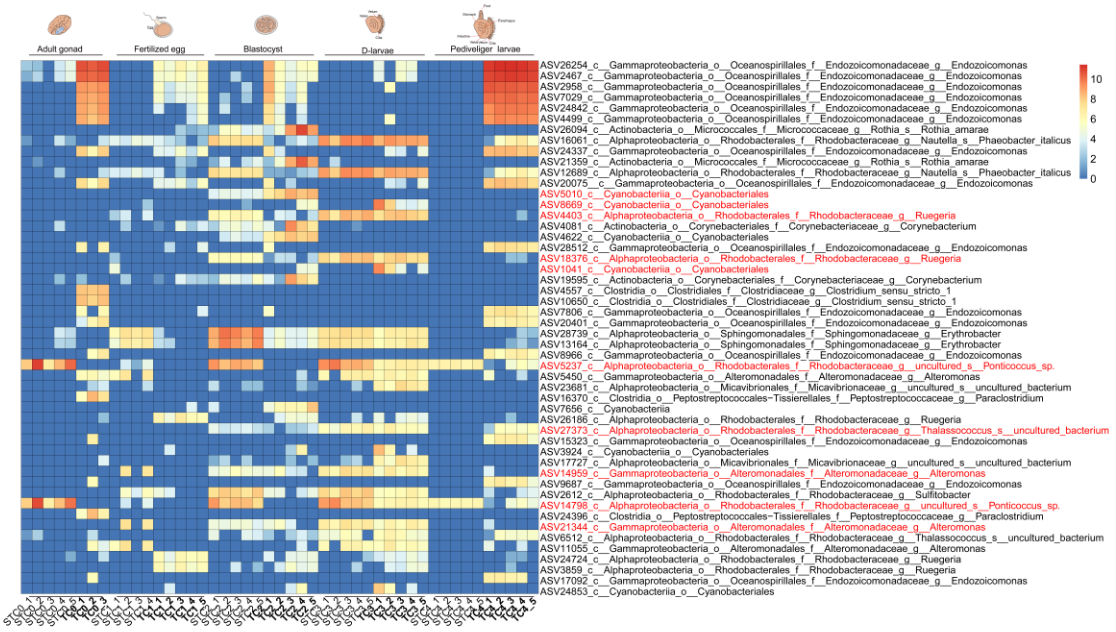

Figure S8. Analysis of top50 ASVs found in giant clam *Tridacna crocea* offspring (data were transformed by log2). The "S" added before the biological sample represents the seawater sample of the corresponding stage. The ASVs highlighted in red signify that initially absent in adult gonads and fertilized eggs, these ASVs gradually appeared as the host developed in both seawater and biological samples. TC0: adult gonad, TC1: fertilized egg, TC2: blastocyst, TC3: D-larvae, TC4: pediveliger larvae. The "S" added before the biological sample represents the seawater sample of the corresponding stage.

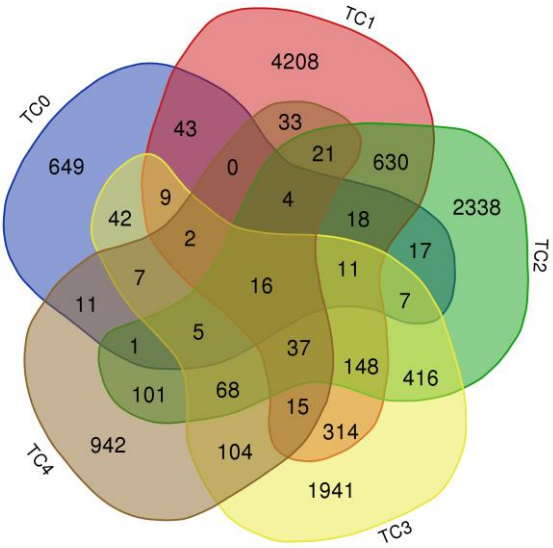

Figure S9. Venn diagrams showing the number of ASVs that were unique or shared in *Tridacna crocea* at each development stage. TC0: adult gonad, TC1: fertilized egg, TC2: blastocyst, TC3: D-larvae, TC4: pediveliger larvae.

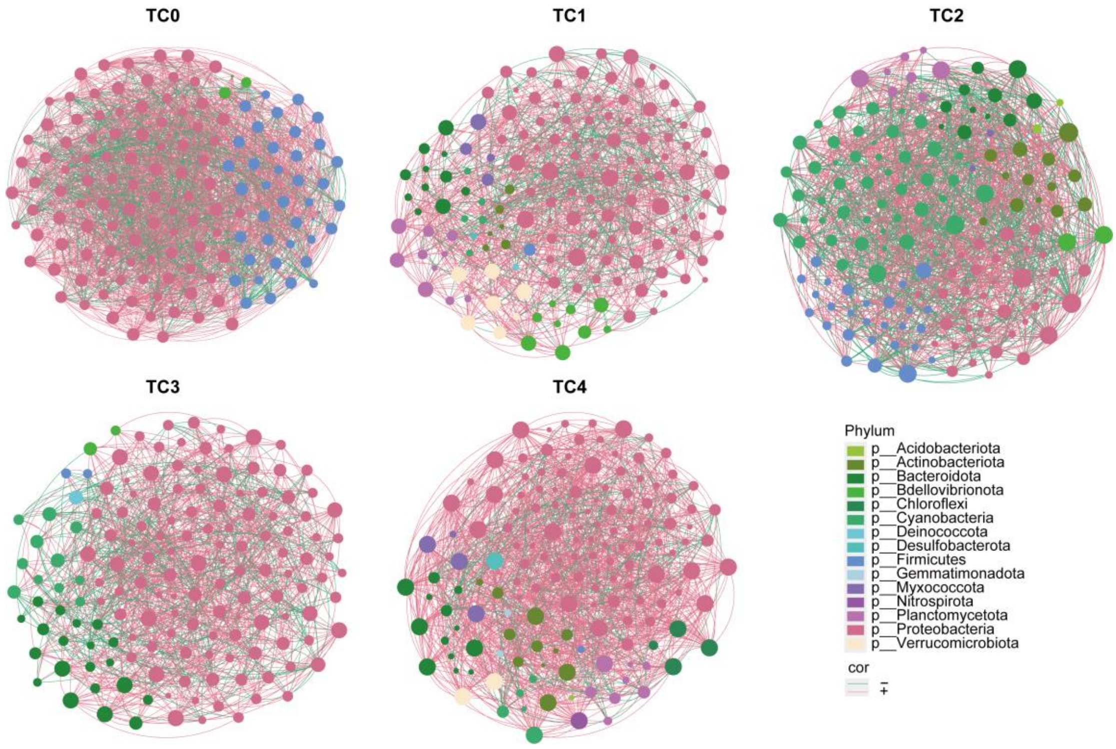

Figure S10. Microbial co-occurrence networks in *Tridacna crocea* at the different life stages. Red lines represent positive correlations, blue lines represent negative correlations, and nodes with the same color belong to the same phylum. TC0: adult gonad, TC1: fertilized egg, TC2: blastocyst, TC3: D-larvae, TC4: pediveliger larvae.

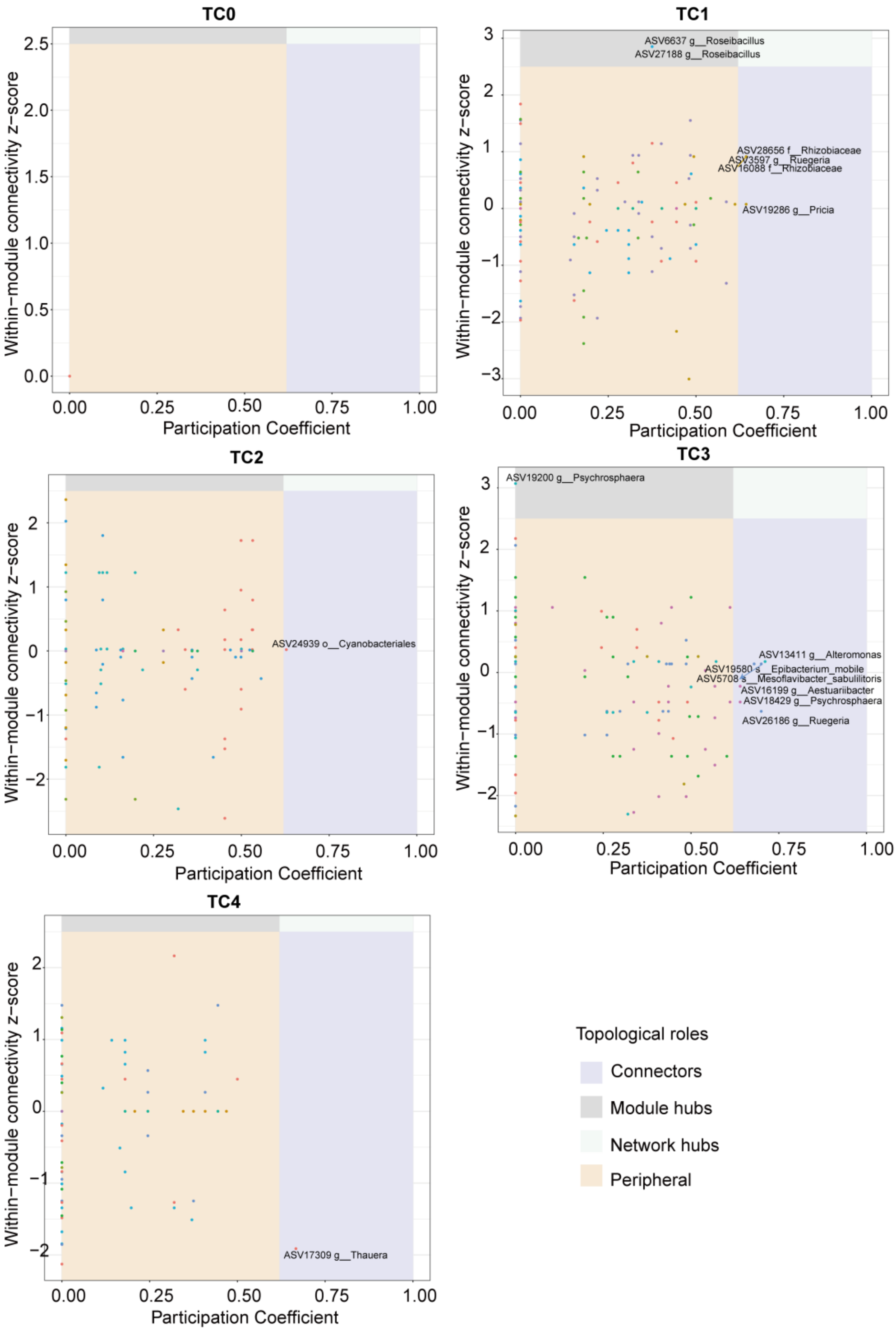

Figure S11. Zi-Pi diagram of bacterial community network at different developmental stages of *Tridacna crocea*. TC0: adult gonad, TC1: fertilized egg, TC2: blastocyst, TC3: D-larvae, TC4: pediveliger larvae.

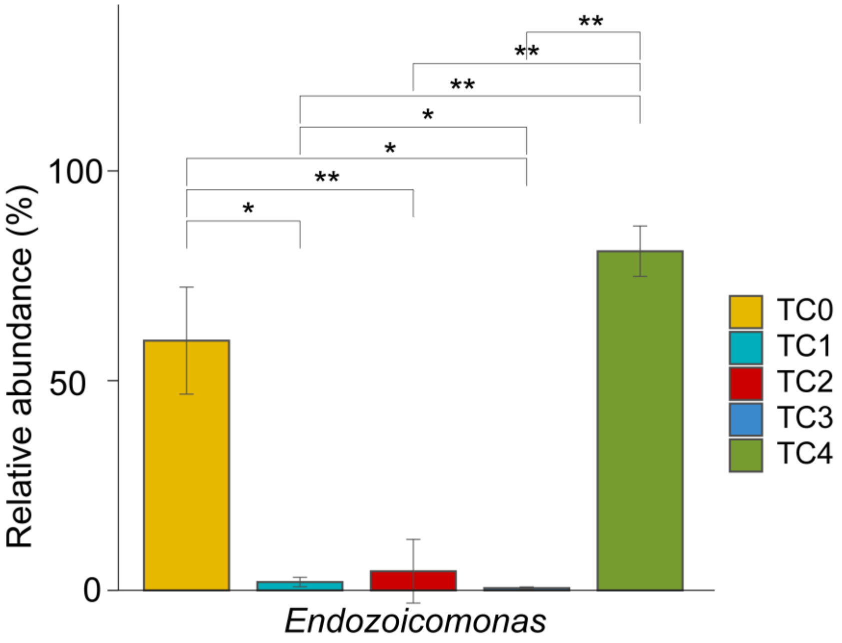

Figure S12. The relative abundance of the genus *Endozoicomonas* in each developmental stage. TC0: adult gonad, TC1: fertilized egg, TC2: blastocyst, TC3: D-larvae, TC4: pediveliger larvae. * $P < 0.05$, ** $P < 0.01$ (T-test).
