# Supplementary material for: Horizontal transmission of symbiotic bacteria and host selective sweep in the giant clam Tridacna crocea
Source: ISME Commun. 2025 Mar 2;5(1):ycaf037. doi: 10.1093/ismeco/ycaf037 (PMC11919647; doi:10.1093/ismeco/ycaf037)
Supplement: Supplementary_methods_ycaf037 [file supplementary_methods_ycaf037.pdf]

Supplementary Information

Materials and methods

Giant clam spawning, rearing, and sample collection

Sufficient oxygen was softly provided using an oxygen machine, and the incubation density was meticulously maintained within 15 cells/mL. After the blastocyst stage (5 h 30 min), approximately 29 h post-fertilization, D-larvae were optimized using a 300-mesh screen. The selected D-shaped larvae were then continued to be kept in filtered seawater (FSW) pools until they developed into the pediveliger larvae (175 h). During the larval cultivation period, the dead larvae were removed, and 12 L of *Isochrysis galbana* (5000 ~ 10,000 cells/mL) was provided twice per day when D-larvae started to filter feeding. The FSW in the incubation tank was exchanged at a rate of 50% ~ 60% daily to ensure sufficient nutrient concentrations. The light intensity is maintained at 4000-5000 lx, the seawater temperature is kept at 27 ± 1 °C, and the salinity is controlled at 32-33‰ to closely mimic natural habitat conditions.

Histology and fluorescence in situ hybridization (FISH) microscopy on slides

For FISH [1, 2], the samples (gonads, fertilized eggs, and pediveliger larvae) that had been embedded in paraffin were sectioned to a thickness of 4 μm. To reduce autofluorescence, the gonadal sections were treated with an autofluorescence eliminator reagent (Merck Millipore, 2160) for 17 h. Subsequently, the sections were treated consecutively with dewaxing, dehydration, air-drying, and immersion in a 0.2 M HCl solution for 12 min, followed by a 10-min treatment with 20 mM Tris-HCl solution (pH 8.0) for 10 min. The sections were then subjected to digestion with proteinase K (10 μg/mL) in 20 mM Tris-HCl solution (pH 8.0) at 37 °C for 10 min and rinsed three times with 20 mM Tris-HCl (pH 8.0) for 5 min each. The section samples were incubated with 5 ng/mL of probe in a hybridization buffer (30% v/v formamide, 0.9 M NaCl, 20 mM Tris-HCl [pH 8.0], 0.01% SDS) at 46 °C for 3.5 h. Hybridizations on tissue sections were performed using mix EUB338 I-III (5'-Cy3 labeled, 5'-GCT GCC TCC CGT AGG AGT-3', 5'-GCA GCC ACC CGT AGG TGT-3', 5'-GCT GCC ACC CGT AGG TGT-3'), the NONEUB338 (Cy3-labeled, 5'-ACA TCC TAC GGG AGG C-3') served as a negative control [1], and no probe was included. Finally, the tissue sections were washed in a preheated wash buffer (0.112 M NaCl, 20 mM Tris-HCl [pH 8.0], 0.01% SDS, 5 mM EDTA) at 48 °C for 20 min, followed by rinsing twice in 20 mM Tris-HCl (pH 8.0) and once in cold water. After air-drying, the sections were mounted in Fluoroshield™ with DAPI (F6057, Sigma-Aldrich) for subsequent analysis. The fluorescence signal was detected using the 546/10 nm laser covering an emission range of 585/40 nm for Cy3 (red), and the 350/50 nm laser covering an emission of 460/50 nm for DAPI (blue). Micrographs of bacterial communities associated with giant clam tissue sections were captured using a fluorescence microscope (Leica DM 2500).

DNA extraction, amplicon sequencing, and data processing

PCR assays were performed in triplicate for each sample in a 20-μL reaction system, and were combined from each sample. The program was as follows: 95 °C for 3 min, followed by 29 cycles (95 °C for 30 s, 55 °C for 30 s, 72 °C for 45 s), 72 °C for 10 min, followed by storage at 10 °C. The purified and quantified amplicons were sequenced using the Pacbio Sequel II System (Pacific Biosciences, CA, USA). Demultiplexed circular consensus sequences (CCS) were generated from PacBio raw reads using the SMRTLink analysis software (version 8.0). The optimized-CCS reads were denoised and processed into amplicon sequence variants (ASVs) using DADA2 [3], where

sequences with 100% similarity are grouped into a single ASV. Denoising to ASVs, taxonomic classification, and filtering of contaminants and rare ASVs were performed using QIIME2 (version 2022.11.1) [4]. Taxonomic assignments were performed using the SILVA reference dataset (v.138). The details of the pipeline are provided in the attached script.

References

1 Wada N, Pollock FJ, Willis BL, Ainsworth T, Mano N, Bourne DG. In situ visualization of bacterial populations in coral tissues: pitfalls and solutions. *PeerJ* 2016;**4**:e2424. https://doi.org/10.7717/peerj.2424

2 Duperron S, Nadalig T, Caprais J-C, Sibuet M, Fiala-Médioni A, Amann R *et al.* Dual symbiosis in a *Bathymodiolus* sp. Mussel from a Methane Seep on the Gabon Continental Margin (Southeast Atlantic): 16S rRNA Phylogeny and Distribution of the Symbionts in Gills. *Appl Environ Microbiol* 2005;**71**:1694-700. https://doi.org/10.1128/AEM.71.4.1694-1700.2005

3 Callahan BJ, McMurdie PJ, Rosen MJ, Han AW, Johnson AJ, Holmes SP. DADA2: High-resolution sample inference from Illumina amplicon data. *Nature methods* 2016;**13**:581-3. https://doi.org/10.1038/nmeth.3869

4 Bolyen E, Rideout JR, Dillon MR, Bokulich NA, Abnet CC, Al-Ghalith GA *et al.* Reproducible, interactive, scalable and extensible microbiome data science using QIIME 2. *Nature biotechnology* 2019;**37**:852-57. https://doi.org/10.1038/s41587-019-0209-9
