# Supplementary material for: Horizontal transmission of symbiotic bacteria and host selective sweep in the giant clam Tridacna crocea
Source: ISME Commun. 2025 Mar 2;5(1):ycaf037. doi: 10.1093/ismeco/ycaf037 (PMC11919647; doi:10.1093/ismeco/ycaf037)
Supplement: Script_ycaf037 [file script_ycaf037.pdf]

```
mkdir demux
cd cleandata
qiime tools import \
--type 'SampleData[SequencesWithQuality]' \
--input-path manifest.txt \
--output-path ../demux/single-end-demux.qza \
--input-format SingleEndFastqManifestPhred33V2
cd ..

qiime demux summarize \
--i-data demux/single-end-demux.qza \
--o-visualization demux/single-end-demux.qzv

mkdir dada2
qiime dada2 denoise-single \
--i-demultiplexed-seqs demux/single-end-demux.qza \
--p-trim-left 0 \
--p-trunc-len 0 \
--o-representative-sequences dada2/rep-seqs.qza \
--o-table dada2/table.qza \
--o-denoising-stats dada2/stats.qza \
--p-n-threads 0

qiime feature-table summarize \
--i-table dada2/table.qza \
--o-visualization dada2/table.qzv \
--m-sample-metadata-file sample-metadata.tsv

mkdir classifier
cd classifier
wget https://data.qiime2.org/2021.11/common/silva-138-99-nb-classifier.qza
cd ..

mkdir taxonomy
qiime feature-classifier classify-sklearn \
--i-classifier classifier/silva-138-99-nb-classifier.qza \
--i-reads dada2/rep-seqs.qza \
--o-classification taxonomy/taxonomy-dada2-sliva.qza \
--verbose \
--p-read-orientation reverse-complement

mkdir filtered_table
qiime feature-table filter-features \
--i-table dada2/table.qza \
```

```
--p-min-frequency 10 \
--o-filtered-table filtered_table/table_filter_low_freq.qza

qiime taxa filter-table \
--i-table filtered_table/table_filter_low_freq.qza \
--i-taxonomy taxonomy/taxonomy-dada2-sliva.qza \
--p-exclude mitochondria,chloroplast \
--o-filtered-table filtered_table/table_filter_low_freq_contam.qza

qiime feature-table summarize \
--i-table filtered_table/table_filter_low_freq_contam.qza \
--o-visualization filtered_table/table_filter_low_freq_contam_summary.qzv

qiime feature-table filter-samples \
--i-table filtered_table/table_filter_low_freq_contam.qza \
--p-min-frequency 4000 \
--o-filtered-table filtered_table/final_table.qza

mkdir filtered_rep_seqs
qiime feature-table filter-seqs \
--i-data dada2/rep-seqs.qza \
--i-table filtered_table/final_table.qza \
--o-filtered-data filtered_rep_seqs/final_rep_seqs.qza

qiime feature-classifier classify-sklearn \
--i-classifier classifier/silva-138-99-nb-classifier.qza \
--i-reads filtered_rep_seqs/final_rep_seqs.qza \
--o-classification taxonomy/final_taxonomy_sliva.qza \
--verbose \
--p-read-orientation reverse-complement

qiime feature-table summarize \
--i-table filtered_table/final_table.qza \
--o-visualization filtered_table/final_table.qzv
```
